# Identification of the prognostic value of elevated ANGPTL4 expression in gallbladder cancer‐associated fibroblasts

**DOI:** 10.1002/cam4.4150

**Published:** 2021-07-31

**Authors:** Fang‐Tao Wang, Xin‐Ping Li, Mu‐Su Pan, Mohamed Hassan, Wei Sun, Yue‐Zu Fan

**Affiliations:** ^1^ Department of Surgery Tongji Hospital Tongji University School of Medicine Shanghai 200065 P.R. China; ^2^ Department of Surgery Shanghai Tenth People's Hospital Tongji University School of Medicine Shanghai 200072 P.R. China

**Keywords:** ANGPTL4, cancer‐associated fibroblast, gallbladder cancer, prognosis

## Abstract

**Background:**

Cancer‐associated fibroblasts (CAFs) with different gene profiles from normal fibroblasts (NFs) have been implicated in tumor progression. Angiopoietin‐like protein 4 (ANGPTL4) has been shown to regulate tumor angiogenesis and metastasis, and predict poor prognosis. However, the ANGPTL4 expression in CAFs, especially in gallbladder CAFs (GCAFs) and its relationship with patient prognosis is unclear.

**Methods:**

Affymetrix gene profile chip analysis in vitro was performed to detect the different gene expression profiles between GCAFs and NFs. RT‐qPCR, immunohistochemistry, and western blotting were performed to investigate the different expression levels of ANGPTL4 in GCAFs/NFs in vitro and in an in vivo nude mouse model of xenograft tumors. Finally, the ANGPTL4 expression was investigated in the stroma of different lesion tissues of the human gallbladder by immunohistochemistry, especially the expression in GCAFs in vivo by co‐immunofluorescence, and their prognostic significance in patients with gallbladder cancer (GBC) was assessed.

**Results:**

ANGPTL4 was upregulated in both GCAFs in vitro and in the xenograft stroma of nude mice in vivo, and its expression was also significantly upregulated in human GBC stroma co‐localized with the interstitial markers fibroblast secreted protein‐1 and α‐smooth muscle actin. In addition, the elevated ANGPTL4 expression in GCAFs was correlated with tumor differentiation, liver metastasis, venous invasion and Nevin staging, and GBC patients with an elevated ANGPTL4 expression in GACFs were found to have a lower survival rate.

**Conclusions:**

Increased ANGPTL4 expression in GCAFs correlates with poor patient prognosis, which indicates a potential therapeutic target for human GBCs.

## Graphical abstract

ANGPTL4 expression was upregulated in GCAF, which was significantly related to the degree of clinical GBC differentiation, liver metastasis, venous invasion, and Nevin staging, and GBC patients with elevated ANGPTL4 expression in GCAF had lower survival rate.

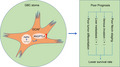

## 1 INTRODUCTION

Gallbladder cancer (GBC) is the most common biliary malignant tumor, with its incidence gradually increasing.^1^, ^2^, ^3^ Since most GBC patients are diagnosed at an advanced stage, surgical resection is not possible. Even if some patients undergo surgery, the tumors will easily recur after surgery. In addition, traditional radiotherapy and chemotherapy can only provide negligible results,^4^, ^5^ which leads to poor prognosis for GBC patients, with a 5‐year survival rate of <5%.^6^ Therefore, the identification of novel prognostic markers and therapeutic targets plays an important role in the development of targeted therapies and in prolonging the survival of GBC patients.

Based on the seed and soil hypothesis for cancer, cancer‐associated fibroblasts (CAFs) are one of the most important components of the tumor microenvironment (TME; i.e., soil),^7^ and CAFs have been found to be related to tumorigenesis, invasion, metastasis, angiogenesis, drug resistance, and prognosis of cancer patients.^8^, ^9^, ^10^, ^11^, ^12^, ^13^ Targeting CAFs can significantly inhibit tumor growth and development, and improve the prognosis of cancer patients.^14^, ^15^, ^16^ However, there are few studies on the specific molecular mechanisms involved. Therefore, it is particularly important to further clarify the ways in which CAFs promote tumor development, especially at the molecular level.

ANGPTL4 (Angiopoietin‐like protein 4) is a novel glycosylated adipokine belonging to the seven ANGPTL protein families, which is known for its involvement in adipokines that regulate lipid and glucose metabolism.^17^, ^18^ A recent study indicated that ANGPTL4 exerts an angiogenic effect by promoting angiogenesis under hypoxic conditions to be involved in the development and progression of cancer.^19^ In addition, recent studies have shown that ANGPTL4 plays a key role in tumor metastasis.^20^, ^21^ Clinical studies have also found that ANGPTL4 expression is significantly associated with vascular and lymphatic invasion in human gastrointestinal tumors, further highlighting the role of ANGPTL4 in tumor metastasis.^22^, ^23^, ^24^ Moreover, ANGPTL4 is a secreted protein that can be detected in the serum, and ANGPTL4 expression in various tumor tissues correlates with patients’ poor prognosis.^25^, ^26^, ^27^ The collective data demonstrated that ANGPTL4 can be used as a potential marker for the prognosis of cancer patients. However, the expression of ANGPTL4 in CAFs, especially in gallbladder CAFs (GCAFs), and its relationship with GBC patient prognosis has not yet been well investigated.

In the present study, we confirmed that ANGPTL4 was upregulated in GCAFs in vitro and the xenograft stroma of nude mice in vivo, as well as in human GBC stroma co‐localized with the interstitial marker fibroblast secreted protein (FSP‐1) and α‐smooth muscle actin (α‐SMA); Patients with elevated expression of ANGPTL4 in GBC stroma had relatively short survival time. We, therefore, hypothesized that the elevated ANGPTL4 expression in GCAFs could be a significant marker for predicting the poor prognosis in GBC patients and discovering the potential targeted therapeutics for human GBCs.

## 2 MATERIALS AND METHODS

### 2.1 Reagents and antibodies

DMEM/F‐12 medium and fetal bovine serum (FBS) were purchased from Gibco. TRIzol reagent and goat anti‐mouse secondary antibody were purchased from Thermo. Primary antibodies against ANGPTL4, α‐SMA, and FSP‐1, and secondary antibody IgG, goat anti‐rabbit secondary antibody, and β‐actin antibody were purchased from Abcam. 3,3‐diaminobenzidine (DAB) solution was purchased from Agilent Technologies. Bovine serum albumin was purchased from Beijing Solarbio Science & Technology.

### 2.2 Cell cultures

Human GBC cell line GBC‐SD was purchased from the Shanghai Cell Biology Research Institute of Chinese Academy of Sciences. Human GCAFs and NFs were originally cultured from three cases of clinical specimens obtained from Tongji Hospital affiliated to Tongji University in 2009, and were identified by detecting interstitial‐specific markers fibroblast activation protein and α‐SMA by western blotting, immunostaining, and co‐immunofluorescence (CIF). The three clinical specimens are from patients with gallbladder adenocarcinoma. They are 46‐year‐old male patient with moderately differentiated and UICCC stage I GBC, 67‐year‐old female patient with moderately differentiated and UICC stage II GBC, and 55‐year‐old female patient with poorly differentiated and UICC stage IIIb GBC. All cells as well as co‐cultures of GCAFs or NFs with GBC‐SD cells (1.5:1) were maintained in DMEM/F‐12 medium supplemented with 10% FBS incubated in a humidified 5% CO_2_ incubator at 37℃.

### 2.3 Affymetrix chip analysis of GCAFs and NFs gene expression profiles in vitro

In order to eliminate the influence of different patients’ own genetic background on the determination of experimental results, we take the GCAFs1 and NFs1 cells of the first patient with primary GBC and take the NFs2 cells of the second patient with primary GBC, that is, a total of three microarrays, and perform gene microarray analysis, respectively, to analyze the upregulated or downregulated genes of GCAFs1/NFs1 and GCAFs1/NFs2, and finally take the intersection result of the two.^28^ Following total RNA quality detection, the cRNA generated by the reverse transcription of RNA and in vitro transcription was then synthesized, purified, and labeled. Affymetrix chip (Affymetrix GeneChip Human 1.0ST array, Affymetrix) was used to investigate the gene expression profile between GCAFs and NFs. Fold change (FC) value >1.5 (*p* < 0.05) was used as the critical value of gene expression. Relevant upregulated genes involved in biological processes performed by Gene Ontology (GO) analysis were further verified.

### 2.4 RNA expression analysis in vitro

Total RNA was extracted from GCAFs or NFs using TRIzol reagent. To detect the ANGPTL4 expression at the mRNA level, qRT‐PCR was performed using real‐time PCR instrument (Thermo) according to the manufacturer's instructions. The specific primers were as follows: ANGPTL4 forward primer: 5’‐GACCTCCGCAGGGACAAGA‐3’; and reverse primer, 5’‐GCTCCGCCCAGATACCATT‐3’; GAPDH forward primer: 5’‐CTCCTCCTGTTCGACAGTCA‐3’; and reverse primer: 5’‐GTTAAAAGCAGCCCTGGTGA‐3’. The mRNAs expression of ANGPTL4 was normalized to GAPDH and was measured by the 2^−ΔΔCq^ method.^29^

### 2.5 Tumor xenograft assay in vivo

Animal experiments were performed following approval and in accordance with the Research Ethical Review Broad of Tongji University. Male BALB/c nude mice (4 week old) were purchased from the Shanghai Laboratory Animal Center of the Chinese Academy of Sciences and housed in specific‐pathogen‐free conditions. The temperature of the culture environment was 26–28℃ and the humidity was 40%–60%. And the nude mice were kept under artificial light for 10 h a day, fed with sterilized and purified food and water. ~1.0 × 10^6^ co‐cultures of GBC‐SD cells/GCAFs or NFs were subcutaneously injected into the right axial back of nude mice when they are weighing 18–20 g. After 1 week, the maximum (a) and minimum (b) diameter of the xenograft in two groups (GBC‐SD + GCAFs and GBC‐SD + NFs; four per group) were measured twice a week, and tumor volumes were calculated using the following formula: *V* = π/6*xab* ^2^. Before it grows to the maximum allowed tumor volume of 1500 mm^3^, mice were sacrificed by CO_2_ (30%) euthanasia after 7 weeks and the tumors were measured and photographed. Xenograft specimens were then used for further immunostaining and western blotting.

### 2.6 Patients and tissue specimens

One hundred and five paraffin‐embedded gallbladder lesion tissue specimens were collected, including 85 GBC samples, 10 specimens of gallbladder precancerous lesions (Adenoma and severe dysplasia; GBPL), and 10 specimens of benign gallbladder lesions (Cholecystitis; GBBL). The study was conducted based on the ethical standards of the Declaration Helsinki, and was approved by the Tongji Hospital Ethics Committee. Written informed consent was obtained from all patients. All the GBC patients who underwent surgery at Tongji Hospital affiliated to Tongji University from January 2007 to September 2012 were histopathologically diagnosed without chemotherapy or radiotherapy prior to surgery. Two independent pathologists uninformed about the patient's clinical condition confirmed the diagnosis of these gallbladder lesion tissue samples. The follow‐up data were completed by telephone survey and the median follow‐up time for GBC patients was 15.0 months, and the 5‐year overall survival was 8.2% (8/85). The collected characteristics of all patients are summarized in Table 1.

**TABLE 1:** Clinicopathologic characteristics of patients with GBC and relationship between ANGPTL4 expression in GBC stroma and the clinical parameters of patients with GBC.

Variable	Patients *n* (%)		ANGPTL4, *n* (%)			*x*^2^ value	*p* value
			Low	High			
Sex							
Male	29 (34.1)		10 (34.5)		19 (65.5)	0.013	0.910
Female	56 (65.9)		20 (35.7)		36 (64.3)		
Age (years)							
>65	45 (52.9)		16 (35.6)		29 (64.4)	0.003	0.957
≤65	40 (47.1)		14 (35.0)		26 (65.0)		
Tumor size (cm)							
>3	49 (57.6)		21 (42.9)		28 (57.1)	2.898	0.089
≤3	36 (42.4)		9 (25.0)		27 (75.0)		
Tumor location							
Bottom	42 (49.4)		12 (28.6)		30 (71.4)	1.643	0.200
Corporis and others		43 (50.6)	18 (41.9)		25 (58.1)		
Histological type							
Adenocarcinoma		79 (92.9)	28 (35.4)		51 (64.6)	0.011	0.917
Other^a^	6 (7.1)		2 (33.3)		4 (66.7)		
Differentiation degree							
G1 (High)	13 (15.3)		8 (61.5)		5 (38.5)	9.714	0.008^b^
G2 (Moderate)	31 (36.5)		14 (45.2)		17 (54.8)		
G3 (Poor)	41 (48.2)		8 (19.5)		33 (80.5)		
Liver metastasis							
(+)	46 (54.1)		11 (23.9)		35 (76.1)	5.686	0.017^b^
(−)	39 (45.9)		19 (48.7)		20 (51.3)		
Venous invasion							
(+)	57 (67.1)		14 (24.6)		43 (75.4)	8.728	0.003^b^
(−)	28 (32.9)		16 (57.1)		12 (42.9)		
Lymph node metastasis							
(+)	56 (65.9)		20 (35.7)		36 (64.3)	0.013	0.910
(−)	29 (34.1)		10 (34.5)		19 (65.5)		
Nevin staging							
S3–S5	74 (87.1)		23 (31.1)		51 (68.9)	4.444	0.035^b^
S1–S2	11 (12.9)		7 (63.6)		4 (36.4)		
Curability							
R1, R2	46 (54.1)		15 (32.6)		31 (67.4)	0.317	0.574
R0	39 (45.9)		15 (38.5)		24 (61.5)		

^a^ Mucinous adenocarcinoma, squamous cell carcinoma, adenosquamous carcinoma.

^b^ *p *< 0.05: statistically significant; GBC, gallbladder cancer.

### 2.7 Immunostaining in vitro and in vivo

For immunostaining in vitro in the co‐culture system of GBC‐SD cells with GCAFs/NFs, cells were treated as described in the previous study.^30^ Then samples were incubated sequentially with primary rabbit anti‐ANGPTL4, secondary antibody IgG, and DAB solution before examined microscopically. For immunostaining in vivo in the xenografts of nude mice obtained from the injected cells with GBC‐SD and CAFs/NFs and in gallbladder lesion tissue specimens, sections were deparaffinized and endogenous peroxidases were inactivated, then the sections were blocked with bovine serum albumin and incubated with antibody against ANGPTL4 overnight at 4℃, followed by an anti‐rabbit IgG secondary antibody and DAB solution. They were then counterstained with hematoxylin.

The staining index (SI) was used to score the immunostaining of ANGPTL4 expression in different gallbladder lesion tissue specimens. SI was calculated from (score of positive cells percentage) × (score of staining intensity). The percentage of positive cells was scored from 0 to 4: 0, no positive cells; 1, 1%–25% positive cells; 2, 26–50% positive cells; 3, 51%–75% positive cells; 4, 76%–100% positive cells. The staining intensity ranged from 0 to 3: 0, negative; 1, weak; 2, moderate; 3, strong. The SI score of 3 was used as the boundary between low protein expression (SI ≤ 3) and high protein expression (SI > 3).

### 2.8 Western blotting

Total protein was extracted from cells or tumor xenografts and quantified with a BCA protein assay kit (Beyotime Biotechnology). Proteins were separated by SDS‐PAGE (sodium dodecyl sulfate‐polyacrylamide gel electrophoresis) and transferred to a PVDF membrane (EMD Millipore) and then was incubated with ANGPTL4 primary anti‐rabbit and β‐actin antibodies, followed by an appropriate secondary anti‐rabbit IgG. The target proteins were visualized using an enhanced chemiluminescent reagent (EMD Millipore), and the band density was quantified using the ImageJ software (National Institutes of Health). When the final xenograft tissue was obtained, a part of each specimen was used to embed paraffin for immunohistochemical staining, and the other part was used to extract proteins for western blotting.

### 2.9 CIF staining in vivo

GBC sections were deparaffinized and pretreated as described for immunostaining. Sections were incubated with rabbit anti‐ANGPTL4 and mouse anti‐α‐SMA/FSP‐1 at 4˚C overnight for CIF staining of ANGPTL4 and α‐SMA/FSP‐1. Then sections were washed in phosphate buffer saline (PBS) and incubated with the following secondary antibodies: goat anti‐rabbit secondary antibody to detect the ANGPTL4 expression, goat anti‐mouse to detect the α‐SMA or FSP‐1 expression. Sections were finally washed in PBS, countered with diamidino phenylindole (DAPI), and observed by immunofluorescence microscopy at 200× magnification.

### 2.10 Statistical analysis

Statistical analysis was calculated by SPSS 22.0 software (IBM Corp) and data are expressed as the mean ± *SD*. Student's *t*‐test and variance test were chosen for comparisons among groups. The Dunn's post hoc test was used to compare ANGPTL4 expression in the stroma of different human gallbladder lesion tissue specimens. The correlation between GBC patient clinicopathological characteristics and ANGPTL4 expression was assessed by χ^2^ test or Fisher's exact test. Survival comparisons were performed by log‐rank test. The significance of various parameters for survival was determined by the Cox regression model. Each experiment was repeated at least three times. *p* < 0.05 was considered to be statistically significant.

## 3 RESULTS

### 3.1 ANGPTL4 expression is elevated in GCAFs in vitro

**FIGURE 1:**
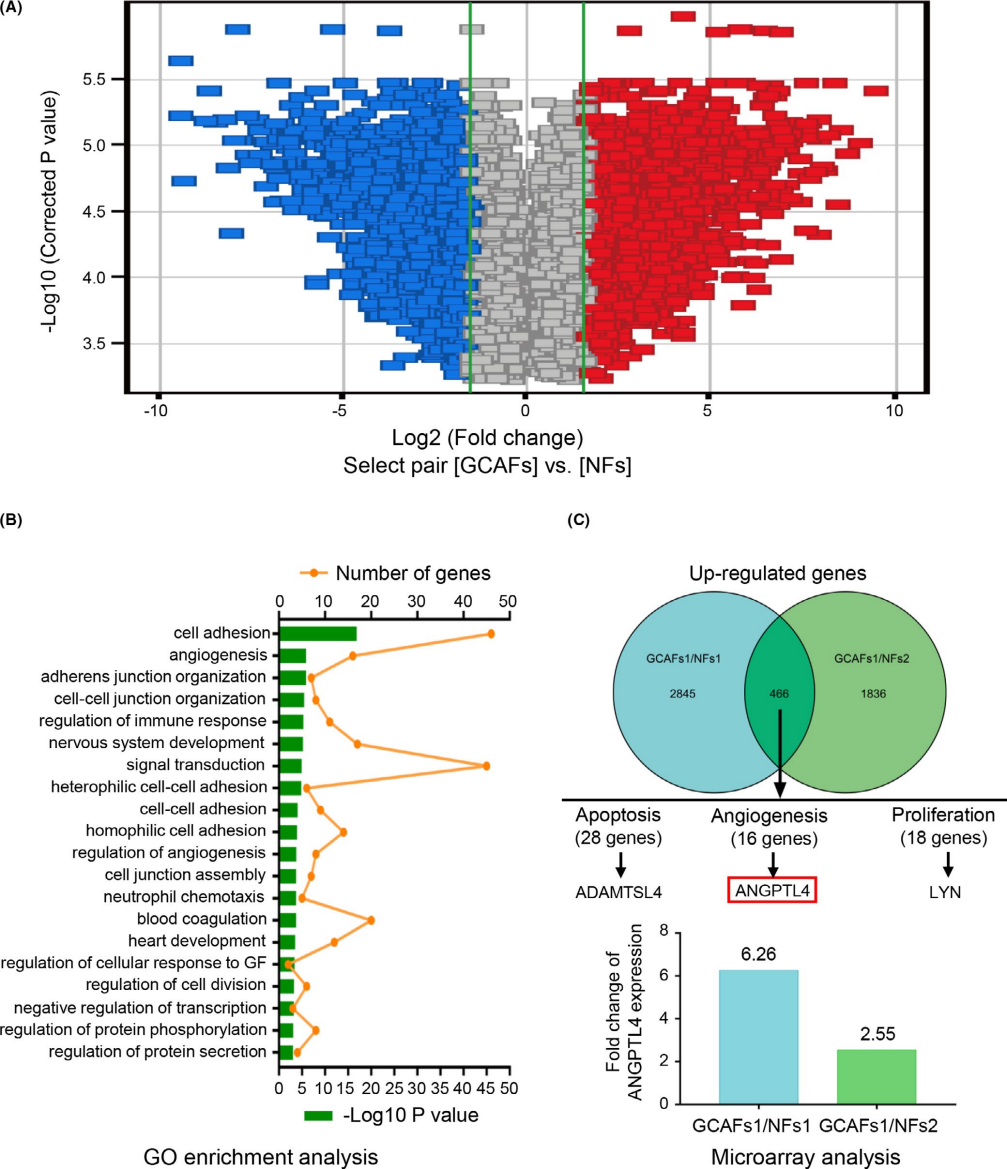
The expression of ANGPTL4 is upregulated in GCAFs in vitro. (A) Gene expression profiles of GCAFs and NFs were identified by Affymetrix GeneChip Human 1.0ST array. Volcano maps depict the genes that were significantly affected in GCAFs and NFs. Downregulated genes are represented in blue, and upregulated genes in red. (B) Gene ontology (GO) enrichment analysis classified genes in selected genesets: the biological processes involved. The top 20 with the most significant differential gene function and the number of genes they contain are listed here. (C) Based on the inclusion criteria, 466 upregulated co‐expression genes (FC > 1.5) were identified between GCAFs and NFs, and the ANGPTL4, as one of the 16 genes related to tumor angiogenesis was significantly upregulated in GCAFs (FC = 4.41)

The Affymetrix GeneChip array was used to identify differentially expressed genes between CAFs and NFs (Figure 1). Volcano maps provided an overview of the genes that are significantly affected (Figure 1A). Gene Ontology (GO) analysis was used to indicate genes that were significantly upregulated, the top 20 with the most significant differential gene function and the number of genes they contain are listed here (Figure 1B). According to the inclusion criteria, 466 upregulated (FC > 1.5) genes were identified between GCAFs and NFs. Among the 16 genes related to angiogenesis, the expression of ANGPTL4 was significantly upregulated in GCAFs (FC = 4.41; Figure 1C).

**FIGURE 2:**
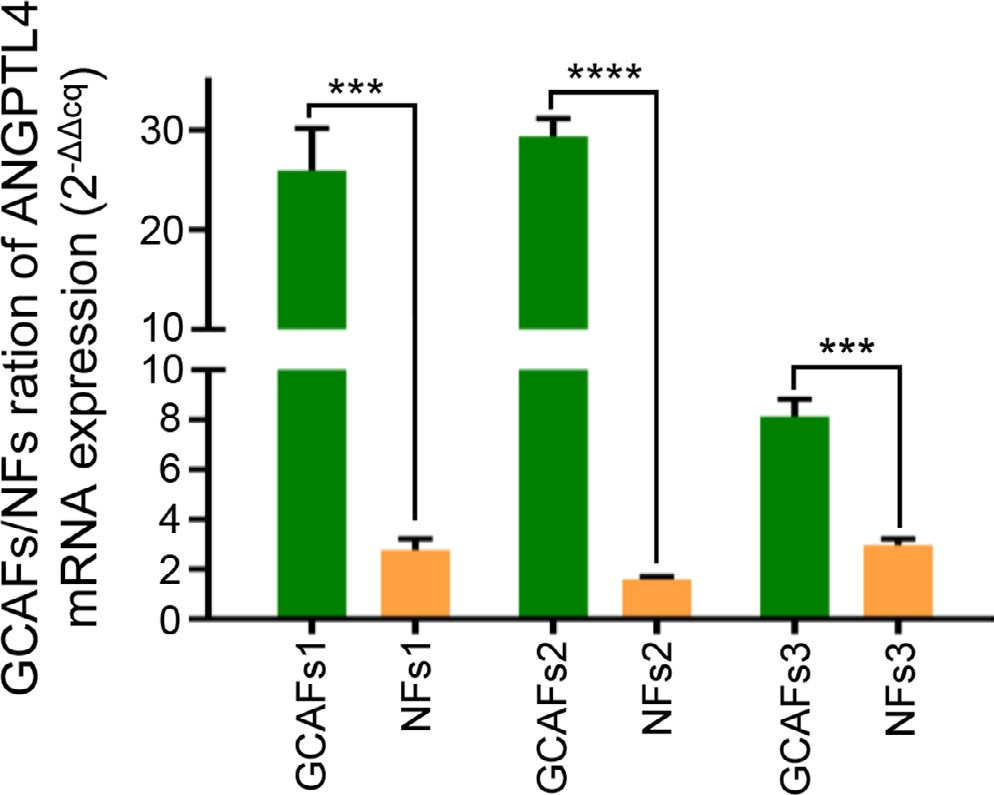
ANGPTL4 RNA expression was detected by RT‐qPCR. Compared with the adjacent gallbladder NFs, the mRNA of ANGPTL4 was significantly upregulated in all GCAFs groups. ^***^ *p* < 0.001 and ^****^ *p* < 0.0001

**FIGURE 3:**
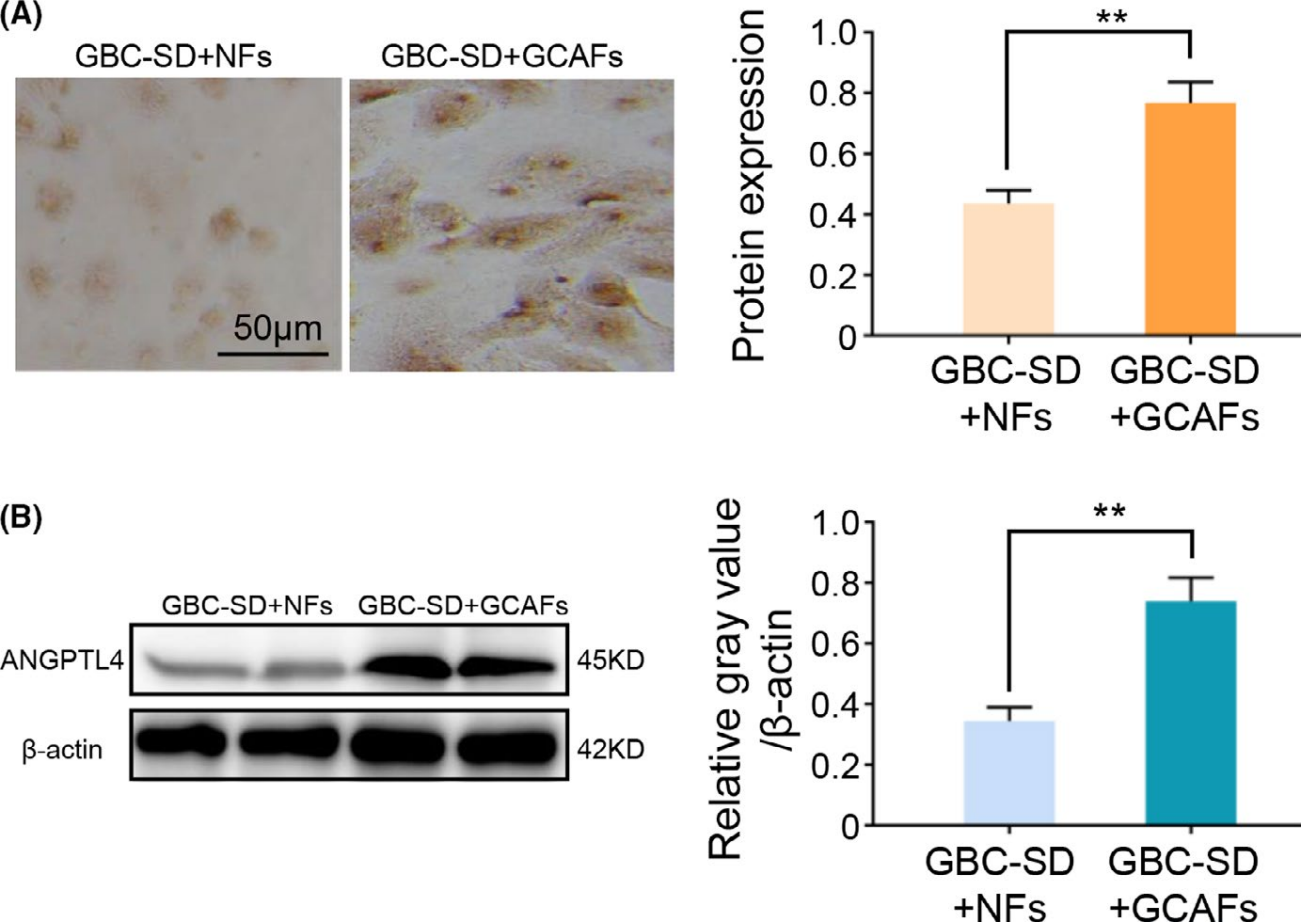
ANGPTL4 expression in the co‐culture of GBC‐SD with GCAFs or NFs in vitro. (A) Immunocytochemical staining of ANGPTL4 expression in a co‐culture system of GBC‐SD + GCAFs/NFs in vitro. (B) Western blotting was used to detect the ANGPTL4 expression in a co‐culture system of GBC‐SD + GCAFs/NFs in vitro. ^**^ *p* < 0.01

In order to further verify the results of gene chip analysis, RT‐qPCR was used to detect the ANGPTL4 expression at the mRNA level in GCAFs and NFs in vitro. These results revealed that ANGPTL4 expression in GCAFs of all samples was significantly increased compared with the adjacent gallbladder NFs (GCAFs vs. NFs; *p *< 0.001 and *p* < 0.0001; Figure 2). Considering that tumor stromal cells interact with tumor cells during tumor growth, the protein expression level of ANGPTL4 was further tested in the co‐culture system of GBC‐SD cells with GCAFs/NFs. As expected, compared with the GBC‐SD + NFs co‐culture, ANGPTL4 protein expression was significantly upregulated in the GBC‐SD + GCAFs co‐culture using immunostaining (0.768 ± 0.068 vs. 0.435 ± 0.045; *p* < 0.01; Figure 3A), which was consistent with the results of western blotting (0.739 ± 0.078 and 0.345 ± 0.045, *p *< 0.01; Figure 3B). In combination, this study showed that the expression of ANGPTL4 was elevated in GCAFs in vitro.

### 3.2 Elevated ANGPTL4 expression in the xenografts of nude mice obtained from the injected cells co‐cultured of GBC‐SD and GCAFs may promote xenograft growth in vivo

**FIGURE 4:**
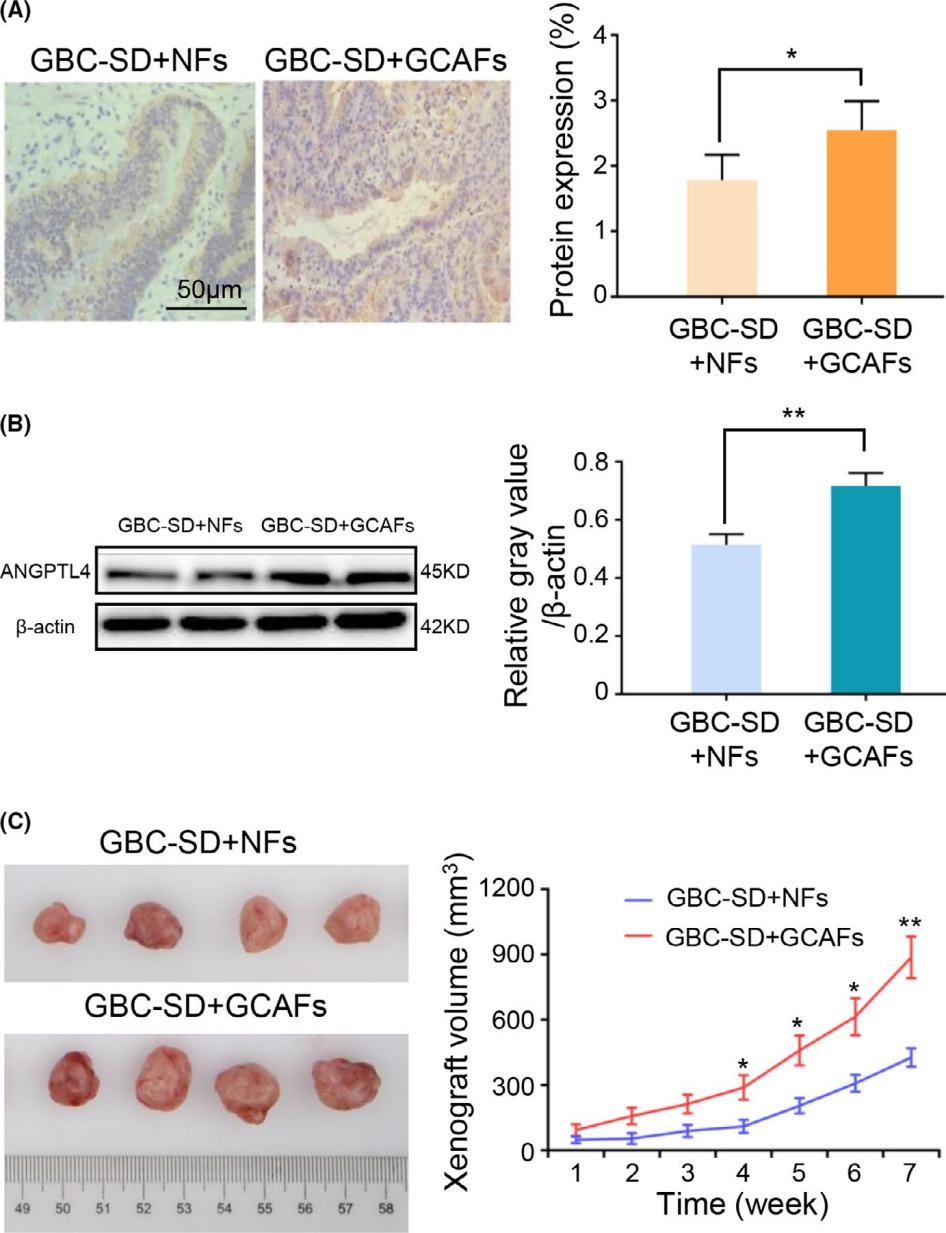
Upregulated ANGPTL4 expression in the xenografts of nude mice obtained from the injected cells co‐cultured of GBC‐SD with GCAFs may promote xenograft growth in vivo. (A) Immunohistochemical staining of ANGPTL4 expression in two groups of the xenografts of nude mice in vivo. (B) Western blotting was used to detect the protein expression of ANGPTL4 in two groups of the xenografts of nude mice in vivo. (C) Tumor xenografts of nude mice obtained from the injected cells co‐cultured of GBC‐SD with CAFs/NFs and the growth curves of the tumor xenografts of each group. The tumor xenograft volume in group GBC‐SD + GCAFs was larger compared with the GBC‐SD + NFs group. ^*^ *p* < 0.05 and ^**^ *p* < 0.01

In order to further detect whether ANGPTL4 is highly expressed in GCAFs in vivo, tumor xenograft model was established by injecting cells co‐cultured of GBC‐SD and GCAFs/NFs. Nude mice were divided into two groups: GBC + GCAFs and GBC + NFs. The xenograft tumors were allowed to develop for 7 weeks following injection. After all mice were sacrificed, the expression of ANGPTL4 in xenografts was detected by immunostaining and western blotting. Compared with the GBC + NFs group, ANGPTL4 expression was significantly upregulated in the GBC + GCAFs group, which was confirmed by both immunostaining (2.545 ± 0.445 vs. 1.786 ± 0.386%; *p* < 0.05; Figure 4A) and western blotting (0.717 ± 0.025 vs. 0.514 ± 0.022; *p* < 0.01; Figure 4B). In addition, the xenograft tumor volume in the GBC + GCAFs group was found to be significantly higher than that in the GBC + NFs group (*p *< 0.05 and *p* < 0.01; Figure 4C). In summary, these data indicated high expression of ANGPTL4 in nude mouse xenografts obtained from the injected cells co‐cultured of GBC‐SD and GCAFs, and we speculated that upregulated ANGPTL4 expression may promote the growth of xenografts in nude mice.

### 3.3 ANGPTL4 is expressed in both α‐SMA‐ and FSP1‐positive fibroblasts in human GBC stroma

**FIGURE 5:**
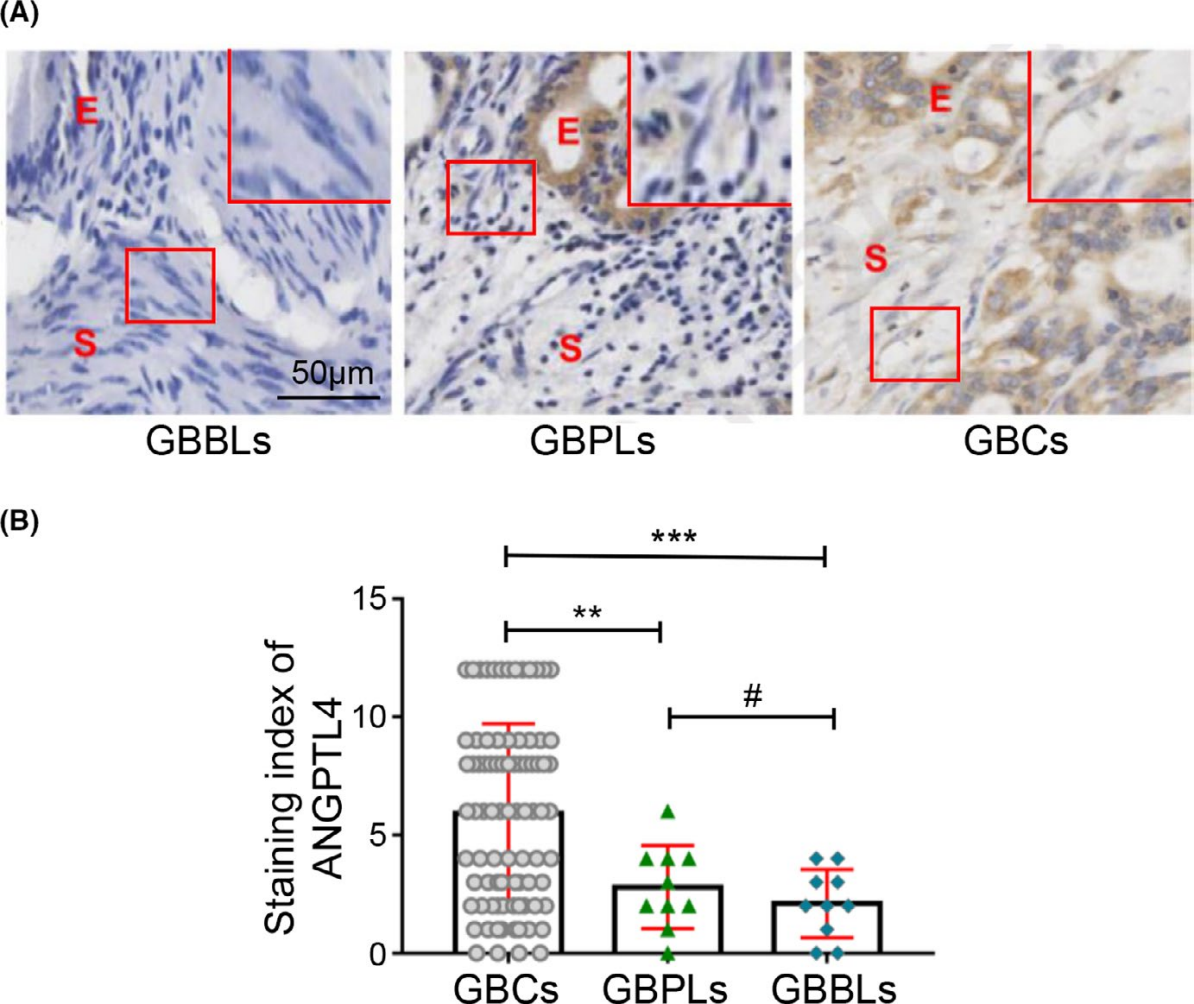
ANGPTL4 expression is upregulated in the stroma of GBCs. (A) ANGPTL4 expression was analyzed by immunohistochemistry staining, E, epithelium; S, stroma. Magnified insets show representative ANGPTL4 staining in stroma. The ANGPTL4 expression in the stroma of GBC (cytoplasm and/or nuclear brown staining) was significantly higher than that in GBPLs (light brown or negative staining) and GBBLs (light brown or negative staining). (B) Immunohistochemistry staining for ANGPTL4 expression in the stroma was scored using SI. ANGPTL4 expression in stroma of GBCs was significantly higher than that in GBPLs stroma (5.918 ± 0.412 vs. 2.800 ± 0.554, **p < 0.01) or GBBLs stroma (5.918 ± 0.412 vs. 2.100 ± 0.458, ***p < 0.001). ANGPTL4 expression in GBPLs stroma was higher than GBBLs stroma, however, the difference was not significant (2.800 ± 0.554 vs. 2.100 ± 0.458, #*p* > 0.05)

Finally, the protein level of ANGPTL4 in the human GBC specimen stroma was analyzed to determine the clinical significance of ANGPTL4 expression. Immunostaining was used to analyze the ANGPTL4 expression in different types of gallbladder tissues (GBCs, GBPLs, and GBBLs; Figure 5A). The results showed that the SI value of the ANGPTL4 expression was significantly higher in GBCs stroma compared with GBPLs (5.918 ± 0.412 vs. 2.800 ± 0.554, *p* < 0.01) or GBBLs (5.918 ± 0.412 vs. 2.100 ± 0.458, *p* < 0.001). The SI value of the ANGPTL4 expression in GBPLs stroma was higher than GBBLs stroma, however, the difference was not significant (2.800 ± 0.554 vs. 2.100 ± 0.458, *p* > 0.05) (Figure 5B). In conclusion, these data indicated higher intensity of ANGCTL4 expression in GBCs stroma, as compared with that in the GBPLs stroma and GBBLs stroma.

**FIGURE 6:**
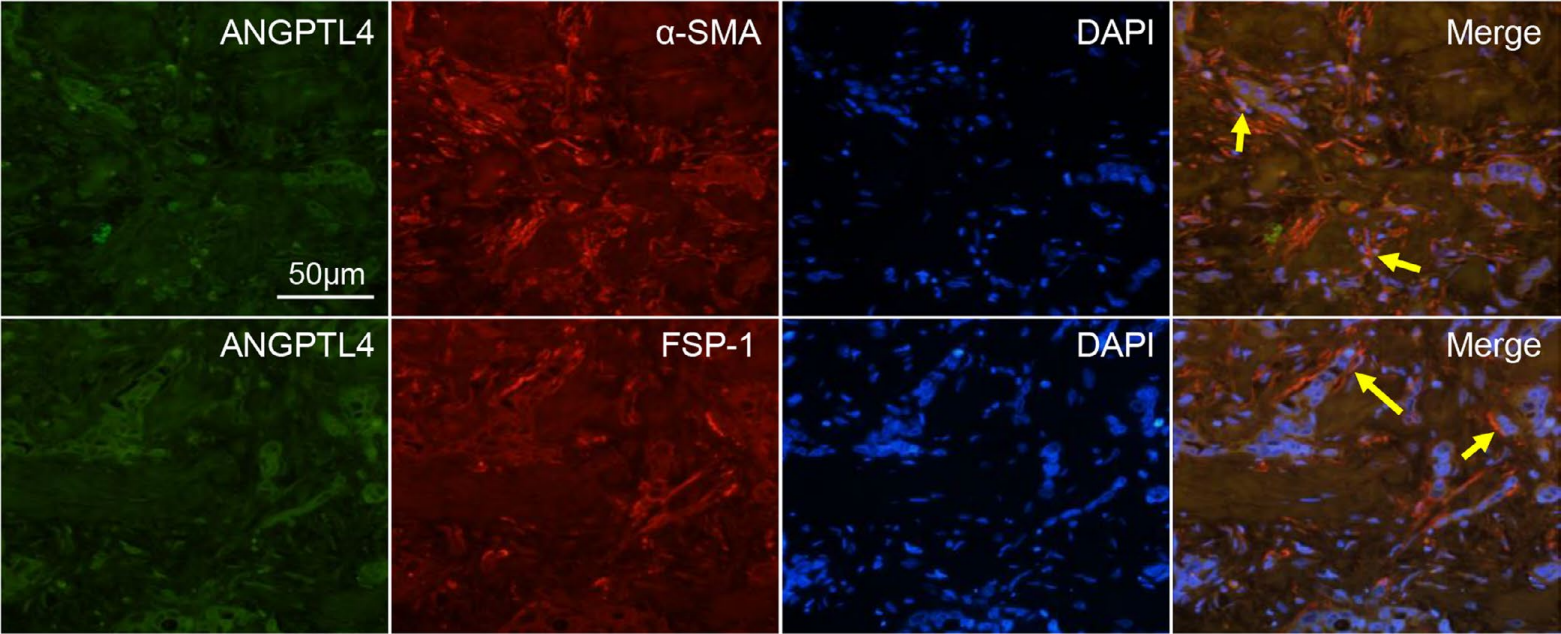
ANGPTL4 expression is co‐localized with both α‐SMA‐ and FSP‐1‐positive stroma. Co‐immunofluorescence staining was performed on GBC patient samples to analyze the expression of ANGPTL4 (green) and α‐SMA or FSP‐1 (red). Arrows show positive staining in stromal fibroblasts

To further determine whether ANGPTL4 was expressed in particularly GCAFs, we performed CIF staining to localize the ANGPTL4 expression with specific markers of fibroblasts, namely α‐SMA or FSP‐1 in GBC stromal tissues. We observed that ANGPTL4 (green) overlapped with cells expressing α‐SMA and FSP‐1 (red) (i.e., GCAFs) (Figure 6), proving that the expression of GCAF‐derived ANGPTL4 was increased in the stroma of human GBCs.

### 3.4 Elevated ANGPTL4 expression in GCAFs is associated with unfavorable prognosis in GBC patients

The correlation between ANGPTL4 expression and GBC patient clinicopathological parameters was shown in Table 1. The results showed that the ANGPTL4 protein was highly expressed in 55 cases (64.7%) in GBC stromal cells and poorly expressed in 30 cases (35.3%). In addition, the ANGPTL4 expression in GBC stromal cells was significantly associated with tumor differentiation (*p* < 0.01), liver metastasis (*p* < 0.05), venous invasion (*p* < 0.01), and Nevin staging (*p* < 0.05), while it was not correlated with patient's sex, age, tumor location, tumor size, histological type, lymph node metastasis, and curability (all *p* > 0.05; Table 1).

**FIGURE 7:**
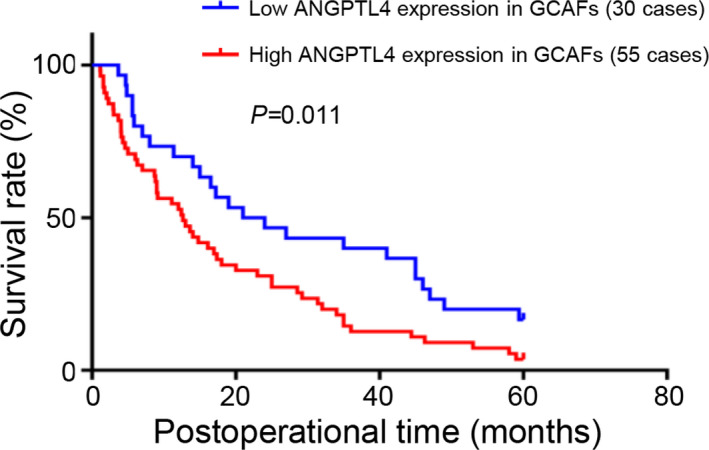
Kaplan–Meier analysis of high and low ANGPTL4 expression in stroma of GBCs. GBC patients with high ANGPTL4 expression in stroma had a shorter survival time compared with patients with low ANGPTL4 expression (log‐rank test; *p* < 0.05)

Furthermore, Cox regression model and log‐rank test were used to determine the relationship between ANGPTL4 expression and prognosis of GBC patients. In a univariate Cox regression analysis, tumor histological type (*p* < 0.01), differentiation degree (*p* < 0.001), liver metastasis (*p* < 0.01), venous invasion (*p* < 0.0001), lymph node metastasis (*p* < 0.05), Nevin staging (*p* < 0.05), curability (*p* < 0.0001), and stromal ANGPTL4 expression (*p* < 0.05) were all significant factors affecting GBC patients’ prognosis. However, in a multivariate Cox regression analysis, only differentiation degree [hazard ratio (HR), 0.437; 95% confidence interval (CI), 0.196–0.975; *p* < 0.05], liver metastasis (HR, 1.949; 95% CI, 1.187–3.201; *p* < 0.01), venous invasion (HR, 2.569; 95% CI, 1.486–4.444; *p* < 0.01), and stromal ANGPTL4 expression (HR, 1.844; 95% CI, 1.098–3.094; *p* < 0.05) were demonstrated as independent prognostic predictors for GBC (Table 2). Moreover, higher levels of ANGPTL4 expression in the GBC stroma were related to a significantly poor survival rate. (Figure 7; *p* < 0.05; log‐rank test). Therefore, these data demonstrated higher intensity of ANGPTL4 expression in human GBC stroma was associated with tumor aggression. Moreover, patients with an elevated ANGPTL4 expression in the GBC stroma have a poorer prognosis.

**TABLE 2:** Analysis of influencing factors of survival prognosis in patients with GBC (Cox model analysis).

Variable (*n*)		Single factor				Multiple factors		
		HR		95% CI	*p* value	HR	95% CI	*p* value
Sex								
Male (29) versus female (56)	0.972			0.609–1.554	0.907			
Age (years)								
>65 (55) versus ≤65 (30)		0.808		0.517–1.262	0.348			
Tumor size (cm)								
>3.0 (49) versus ≤3.0 (36)		1.434		0.909–2.262	0.121			
Tumor location								
Bottom (42) versus corporis and other (43)		0.755		0.481–1.184	0.221			
Histological type								
Adenocarcinoma (79) versus other^a^ (6)			0.255	0.106–0.613	0.002^b^			
Differentiation degree								
G1 (13) versus G2 and G3 (72)		0.243		0.114–0.514	0.000^b^	0.437	0.196–0.975	0.043^b^
Liver metastasis								
(+) (46) versus (−) (39)		2.075		1.294–3.327	0.002^b^	1.949	1.187–3.201	0.008^b^
Venous invasion								
(+) (57) versus (−) (28)		3.235		1.911–5.475	0.000^b^	2.569	1.486–4.444	0.001^b^
Lymph node metastasis								
(+) (56) versus (−) (29)		1.708		1.057–2.762	0.029^b^			
Nevin staging								
S3–S5 (74) versus S1–S2 (11)		2.482		1.227–5.021	0.011^b^			
Curability								
R0 (39) versus R1 and R2 (46)		0.413		1.672–5.041	0.000^b^			
ANGPTL4 in stroma								
High (55) versus low (30)		1.822		1.069–3.104	0.025^b^	1.844	1.098–3.094	0.021^b^

^a^ mucinous adenocarcinoma, squamous cell carcinoma, adenosquamous carcinoma.

^b^ *P* <0.05, statistically significant; HR, risk ratio; CI, confidence interval.

## 4 DISCUSSION

A large number of studies have showed that TME plays a pivotal role in tumorigenesis and progression. CAFs are one of the most important cellular components in the TME, and are reported to regulate the TME balance at the tumor–host interface through variety of ways, such as cell–cell contact, soluble factor secretion; they also promote the malignant transformation of epithelial cells.^9^ CAFs have the characteristics of fibroblasts and smooth muscle cells, express mesenchymal cell markers α‐SMA, fibroblast activation protein, vimentin, FSP‐1, etc., and have been shown in a variety of tumors to have different gene expression profiles than those of NFs.^15^, ^31^, ^32^

ANGPTL4 is a member of the angiogenin family.^33^ ANGPTL4 can be expressed in liver, adipose, placental, and ischemic tissue, and has a strong pro‐angiogenic effect that is independent of the vascular endothelial growth factor (VEGF).^19^, ^34^ Recent studies have shown that angiopoietin plays an important regulatory role in tumor progression, cell growth, and differentiation.^35^, ^36^ ANGPTL4 expression was found upregulated in breast tumors, basal cell carcinoma, melanoma, as well as CRC cells, and cancer cell lines derived from breast, lung, and liver cancers.^37^, ^38^ Moreover, certain studies have confirmed that ANGPTL4 can promote vascular invasion and distant metastasis of gastric cancer and CRC,^22^, ^23^ and transforming growth factor (TGF‐β) can induce ANGPTL4 expression, thereby promoting lung metastasis in breast cancer patients.^39^ Based on tumor clinicopathology, high ANGPTL4 expression is associated with increased oral cancer incidence and poor prognosis.^37^, ^40^ The elevation of ANGPTL4 in CRC is also associated with a shorter disease‐free survival.^38^ And the expression of ANGPTL4 in prostate cancer can be used as a clinical prognostic marker and therapeutic target.^25^

However, these previous studies merely focused on ANGPTL4 expression in tumor cells and it is unclear whether ANGPTL4 is highly expressed in CAFs, one of the most important mesenchymal cells in the TME, and how it relates to the prognosis of tumor patients. TME tends to be hypoxic due to the abnormal growth of tumor cells and poor blood vessel formation. Recent studies have shown that ANGPTL4 expression can be induced under hypoxic conditions, and the upregulation of ANGPTL4 is caused by the transcription factor hypoxia‐inducible factor 1α (HIF‐1α).^38^, ^41^, ^42^ Studies have also shown TGF‐β and HIF‐1α play an important role in the function of CAFs.^43^ CAFs affect the malignant behavior of tumor cells and treatment tolerance through a variety of autocrine or paracrine modes^7^, ^9^, ^13^, ^14^, ^44^ and are associated with poor prognosis^13^, ^45^; anti‐CAFs can effectively prevent tumor progression.^16^, ^46^, ^47^, ^48^ In our study, the different gene expression profiles between GCAFs and NFs were first analyzed using Affymetrix chips, proving that the ANGPTL4 related to tumor angiogenesis was significantly upregulated in GCAFs. It was further confirmed that ANGPTL4 was highly expressed in GCAFs at the mRNA and protein levels in vitro and in nude mouse xenograft stroma in vivo. In addition, ANGPTL4 was positively expressed in human GBC stroma, co‐stained with α‐SMA‐ and FSP‐1‐positive stromal cells, indicating an increased expression of ANGPTL4 in GCAFs. Therefore, it is inferred that the high expression of ANGPTL4 in GCAFs can also promote angiogenesis of GBC, accelerate the growth of GBC cells, and promote vascular infiltration and metastasis. Through further research and analysis, we found that the increased ANGPTL4 expression in the GBC stroma was associated with tumor invasive activity characteristics such as differentiation, liver metastasis, venous invasion, and curability, and ANGPTL4 expression in GCAFs was an independent prognostic factor for GBC patients. Also, patients with a high ANGPTL4 expression in GCAFs had a lower survival time. These findings are consistent with previous studies on the role of ANGPTL4 in promoting tumor development and predicting poor prognosis.

In conclusion, the present study first revealed that the differential gene expression between GCAFs and NFs, and the increased expression of ANGPTL4 at the mRNA and protein levels in GCAFs and GBC + GCAFs in vitro and in nude mouse xenograft stroma in vivo, and that the elevated expression of ANGPTL4 in GCAFs may promote the growth of xenografts in nude mice. Moreover, the expression of ANGPTL4 was also increased in the stroma of human GBCs, and ANGPTL4 expression was derived from α‐SMA‐ and FSP‐1‐positive GCAFs. The elevated ANGPTL4 expression in GCAFs was associated with tumor aggression characteristics, indicating a poor prognosis for GBCs patients. However, the potential molecular mechanism through which the elevated ANGPTL4 expression in GCAFs predicts poor prognosis in GBC patients was not elucidated herein. Another question that was not answered in the present study was whether targeting ANGPTL4 in GCAFs affects the viability of GBC. Further research will help to clarify the underlying molecular mechanisms and provide new targets for individualized treatment of GBCs.

## CONFLICTS OF INTEREST

The authors declare that they have no conflict of interests.

## AUTHORS’ CONTRIBUTIONS

FTW, WS, and YZF designed and coordinated the study. FTW, XPL, and MSP carried out all the experiments involved in the research. FTW, WS, and YZF performed the statistical analysis. XPL, MSP, and MH participated in the collection of reagents/materials/analysis tools. FTW, WS, and YZF drafted the paper. All authors read and approved the manuscript.

## Data Availability

Not applicable.
